# Surgical management of large facial congenital melanocytic nevi using Ahmad technique: a case report

**DOI:** 10.1093/jscr/rjaf612

**Published:** 2025-08-12

**Authors:** Ahmad Fayez Ahmad

**Affiliations:** Department of Oral and Maxillofacial Surgery, Tishreen University Hospital, Al-Kournish Al-Janoubi Street, Al-Mohafaza District, Latakia, Latakia Governorate, 4011, Syria

**Keywords:** congenital melanocytic nevus, tissue expansion, facial reconstruction, Ahmad technique

## Abstract

We report the case of a 9-year-old girl with a large congenital hairy pigmented nevus on her left cheek causing psychological distress. A novel and promising surgical method called the ‘Ahmad Technique,’ named after the author of this manuscript, was employed. This approach uses a single tissue expander in a structured four-stage treatment plan. Initially, a 30 ml subcutaneous expander was inserted via a preauricular incision and gradually inflated every 10 days over 4 months. After complete deflation, a partial excision of the nevus was performed, with histopathology confirming a benign intradermal melanocytic nevus. Following wound healing, tissue expansion resumed for another four months. The third stage included a second partial excision followed by a final expansion phase. In the fourth stage, complete excision of the nevus and expander capsule was done, and reconstruction used the expanded skin flap. The patient achieved excellent aesthetic and psychological outcomes, confirming the Ahmad Technique as a safe, effective, and innovative option for complex facial congenital melanocytic nevi.

## Introduction

Congenital melanocytic nevi (CMN) are benign proliferations of melanocytes that are present at birth and vary in size, location, and appearance. Large or facial CMN can have considerable psychosocial and aesthetic impact, particularly in pediatric patients. Although the lifetime risk of malignant transformation is relatively low, estimated between 1% and 2%, early and planned surgical intervention is often recommended for both preventive and reconstructive purposes [1].

Classification of CMN by using the Periodic Acid–Schiff (PAS) [2]:

- Small CMN: **<** 1.5 cm
- Medium CMN: 1.5–19.9 cm
- Large CMN: **≥** 20 cm
- Giant CMN: **≥** 40 cm or covering ≥2% of total body surface area in neonates.

The selection of a surgical technique depends on the lesion’s size, anatomical location, and patient-specific factors. Small CMN may be effectively treated with direct excision and primary closure [1]. Medium-sized lesions often require serial (staged) excision to allow tissue relaxation between procedures [3]. For larger or cosmetically sensitive lesions, tissue expansion is widely considered the gold standard, offering reconstruction with adjacent skin of matching texture, color, and thickness [4, 5]. When expansion is not feasible, options such as skin grafting [6], local flaps [7], or dermal substitutes [8] may be considered.

## Case presentation

**Figure 1:**
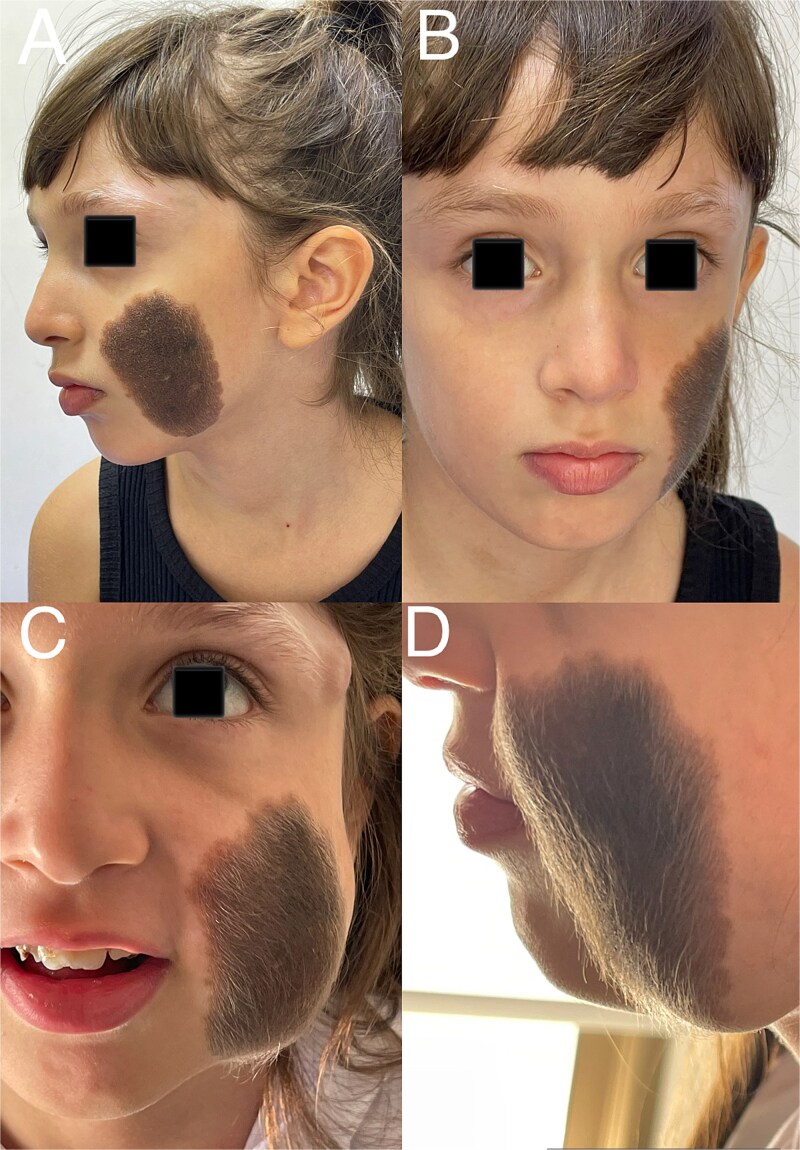
(A) Patient photograph (frontal view). (B) Patient photograph (lateral view). (C) Patient photograph after full expansion of the tissue expander (frontal view). (D) Patient photograph after full expansion of the tissue expander (lateral view).

A 9-year-old girl was referred to our department with a large congenital hairy pigmented nevus involving the left cheek and periorbital area (Fig. 1A and B). The lesion caused significant psychological distress and social withdrawal. Histopathological examinations were performed during the second and third stages of nevus excision, and no signs of malignancy were detected, as the presence of malignancy would have significantly altered the intraoperative surgical plan.

A 30 ml subcutaneous tissue expander was inserted through a preauricular incision, superficial to the superficial musculoaponeurotic system (SMAS) layer, with the injection port positioned in the lateral orbital region. This site was chosen due to limited availability of longer injection tubing in our healthcare setting, as a result of ongoing conflict-related supply constraints. The lateral orbital rim provided a practical and safe alternative that avoided interference with the surgical field and allowed easy access during serial expansions.

This dissection plane was deliberately chosen to minimize the risk of injury to the branches of the facial nerve, especially given the need for multiple surgical interventions in the same anatomical region. Serial saline inflations were performed every 10 days over a 4-month period. The expansion phase was uneventful, and the patient tolerated the procedure well (Fig. 1C and D).

**Figure 2:**
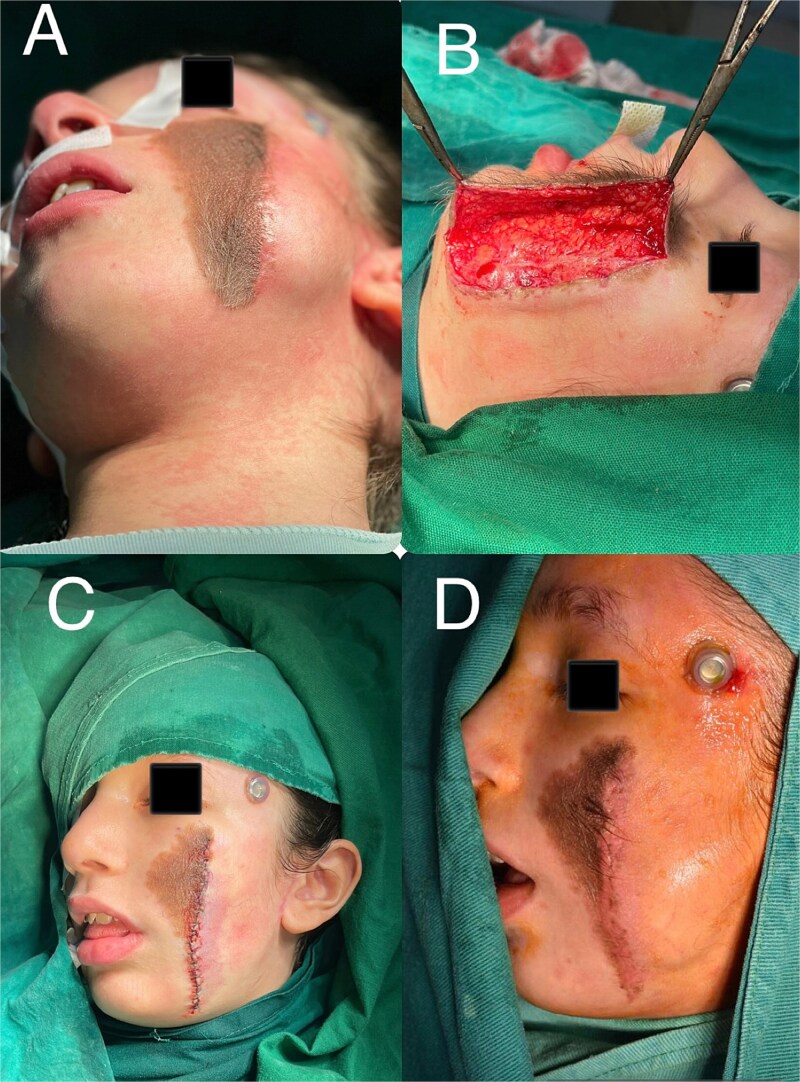
(A) Patient appearance prior to the third surgical stage. (B) Surgical incision site. (C) Operative field at the end of the third surgical procedure. (D) Lateral view of the patient prior to the final surgical stage.

Prior to the second surgical procedure, the expander was fully deflated. Subcutaneous dissection was performed with meticulous care to avoid injury to the expander and surrounding tissues. Approximately 50% of the nevus was excised. In the early postoperative period, partial exposure of the injection port occurred. This was managed conservatively with regular dressing changes and prophylactic antibiotics. Once the wound stabilized, tissue expansion was resumed one month later and continued every 10 days for an additional 4 months (Fig. 2A–C).

**Figure 3:**
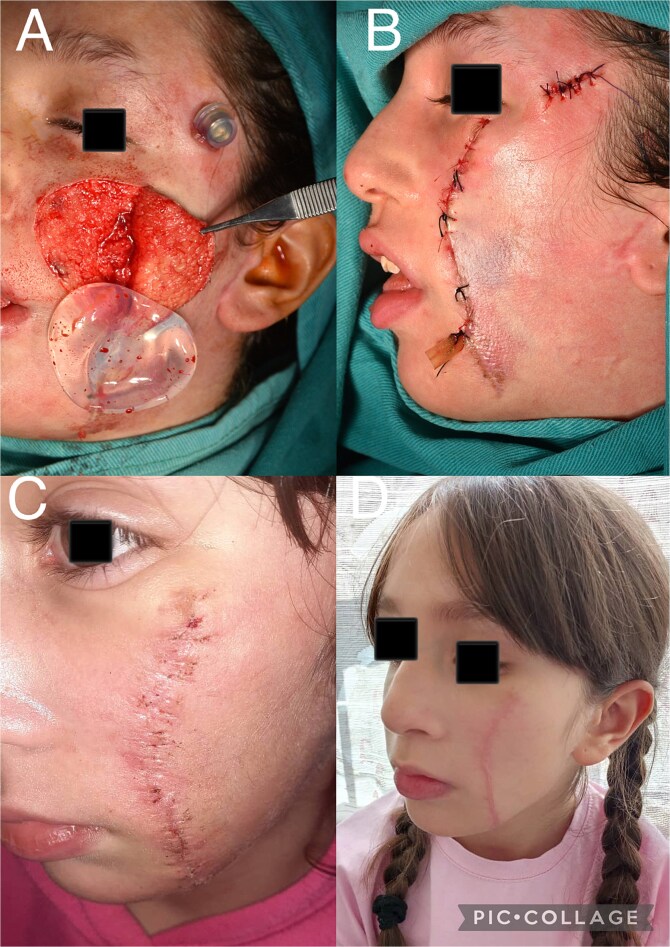
(A) Intraoperative view showing complete excision of the nevus and removal of the tissue expander. (B) Lateral view of the patient at the completion of the final surgical stage. (C) Postoperative view of the patient after removal of surgical sutures. (D) Postoperative view of the patient six months after surgery.

In the final stage, the remaining nevus was completely excised, and the capsule surrounding the tissue expander was entirely removed. The resulting defect was reconstructed using the well-vascularized expanded skin flap (Figs 2D and 3A and B). The follow-up period revealed no evidence of postoperative complications (Fig. 3C and D).

## Discussion

In our case, we successfully managed a large congenital melanocytic nevus (CMN) of the face using a four-stage approach with a single tissue expander throughout all stages. This technique reduced the need for multiple surgical sites or additional expanders, thereby minimizing the risk of complications and the psychological burden on the patient. One complication occurred in the form of partial exposure of the injection port, but there were no signs of infection, and it did not affect the surgical treatment plan in any way.

Our approach contrasts with that of Le *et al.* (2024), who treated a dorsal giant congenital melanocytic nevus (GCMN) using two expanders initially and a third in a subsequent stage, achieving complete excision over six months and three surgeries [9]. Similarly, Tran *et al*. (2024) reported a case involving a hemifacial nevus treated with serial tissue expansion followed by full-thickness skin grafting, combining techniques to achieve both functional and aesthetic goals [10].

Furthermore, a 40-year retrospective study by Lemos *et al.* (2020) described the use of serial excision alone, without tissue expansion, in over 30 patients with GCMN. Although effective, this method required long-term follow-up and multiple procedures extending over several years [11].

The key advantage of our method lies in the use of a single expander throughout the entire treatment course, which simplified management, reduced healthcare costs, and minimized patient discomfort. Despite a minor postoperative complication (partial port exposure), the expander remained viable, and tissue expansion could be resumed after wound stabilization. This case highlights that, with meticulous planning and careful intraoperative handling, it is possible to achieve complete excision and optimal reconstruction using a single expander, even in complex facial lesions.

## Conclusion

The Ahmed technique offers a promising and practical approach for the excision of large congenital facial nevi in children. This technique successfully balances oncologic safety, aesthetic outcomes, and the psychological well-being of the patient. The result was considered highly satisfactory by both the patient's family and the surgical team, further supporting the reliability of this method and encouraging its broader application in clinical practice.
